# Neurocognitive Recovery Following Continuous Positive Airway Pressure Therapy in Patients with Moderate to Severe Obstructive Sleep Apnea

**DOI:** 10.3390/jcm14238319

**Published:** 2025-11-23

**Authors:** Jelena Šarić Jurić, Mirjana Grebenar Čerkez, Željko Zubčić, Silvio Bašić, Ivana Bašić, Sanja Jandrić, Kristina Kralik, Stjepan Jurić

**Affiliations:** 1 Faculty of Medicine Osijek, Josip Juraj Strossmayer University of Osijek, 31000 Osijek, Croatia; jelenasaricjuric@gmail.com (J.Š.J.); mirjanagrebenar@gmail.com (M.G.Č.); zzubcic21@gmail.com (Ž.Z.); sbasic@kbd.hr (S.B.); ibasic@kbd.hr (I.B.); jandric.sanja1@gmail.com (S.J.); kkralik@mefos.hr (K.K.); 2 Department of Neurology, University Hospital Centre Osijek, 31000 Osijek, Croatia; 3 Department of Otorhinolaryngology and Head and Neck Surgery, University Hospital Centre Osijek, 31000 Osijek, Croatia; 4 Department of Neurology, University Hospital Dubrava, 10000 Zagreb, Croatia; 5 School of Medicine, University of Zagreb, 10000 Zagreb, Croatia; 6 Department of Neurology, Neurosurgery and Neuropathology, University of Applied Health Sciences, 10000 Zagreb, Croatia; 7 Unit for Child and Adolescent Psychiatry, University Hospital Centre Osijek, 31000 Osijek, Croatia

**Keywords:** obstructive sleep apnea, continuous positive airway pressure, cognition, event-related potentials, Montreal Cognitive Assessment, P300

## Abstract

**Background/Objectives**: Obstructive sleep apnea (OSA) is a common sleep-related breathing disorder associated with cognitive decline. Evidence regarding the reversibility of these deficits after continuous positive airway pressure (CPAP) therapy remains inconsistent. **Methods**: This prospective observational study included 60 adults with newly diagnosed moderate-to-severe OSA (median age 51 years; 80% male), who underwent baseline assessment using P300 event-related potentials and the Montreal Cognitive Assessment (MoCA). After three months of CPAP therapy with confirmed good compliance, both measures were re-evaluated. **Results**: After three months of CPAP therapy, a statistically significant reduction in P300 latency (*p* < 0.001) and an increase in amplitude were observed, accompanied by significant improvements in executive function (*p* < 0.001), attention (*p* < 0.001), and delayed recall (*p* < 0.001). **Conclusions**: CPAP therapy leads to measurable recovery of cognitive function in patients with moderate-to-severe OSA, suggesting partial reversibility of neurocognitive impairment associated with sleep-disordered breathing. These findings highlight the importance of early recognition and treatment of OSA to prevent long-term cognitive decline.

## 1. Introduction

Obstructive sleep apnea (OSA) represents a major global health concern due to its high prevalence and strong association with multiple systemic and neurocognitive complications. It is a common sleep-related breathing disorder characterized by recurrent episodes of partial or complete upper airway collapse during sleep, resulting in reduced (hypopnea) or absent (apnea) airflow despite ongoing respiratory effort [1]. These events lead to intermittent hypoxemia, sleep fragmentation, and consequent impairment of restorative sleep processes.

Epidemiological studies estimate that OSA, defined by an apnea–hypopnea index (AHI) > 5 events per hour, affects approximately 936 million adults aged 30–69 years worldwide, with nearly 425 million individuals experiencing moderate-to-severe OSA (AHI > 15 events per hour) [2]. The disorder is associated with a broad range of adverse health outcomes, including cardiovascular, cerebrovascular, metabolic, and cognitive impairments [3].

Cognitive dysfunction in OSA commonly manifests as deficits in memory, executive function, attention, vigilance, and visuospatial processing [4,5,6,7,8]. However, the true prevalence and severity of these deficits remain unclear due to methodological heterogeneity and inconsistent findings across studies. The mechanisms underlying cognitive impairment in OSA are not fully elucidated, but intermittent hypoxemia and sleep fragmentation are considered key contributors to structural and functional brain alterations.

Objective evaluation of cognitive function in OSA can be achieved through standardized neuropsychological testing and electrophysiological measures, such as event-related potentials (ERPs), particularly the P300 component. The P300 wave serves as a well-established electrophysiological marker of higher-order cognitive processing, including attention, memory, and information integration [9]. Nevertheless, studies examining ERPs in OSA patients have yielded mixed results: while some report prolonged P300 latency and reduced amplitude, others found no significant differences compared with healthy controls [10,11,12,13,14,15,16,17].

Continuous positive airway pressure (CPAP) therapy is the standard treatment for OSA and has been associated with partial improvement in cognitive functions in some studies, although findings remain inconsistent [18,19,20,21]. The Montreal Cognitive Assessment (MoCA) has also been widely employed to screen for cognitive impairment in OSA patients, frequently revealing deficits across multiple domains—memory, attention, executive function, and visuospatial ability [22]. Some reports suggest that effective CPAP therapy may enhance MoCA performance, indicating partial recovery of cognitive function; however, the degree and consistency of cognitive improvement remain uncertain.

Therefore, the present study aimed to comprehensively evaluate cognitive recovery following CPAP treatment using both electrophysiological (P300) and neuropsychological (MoCA) measures, to provide an integrated assessment of cognitive outcomes in patients with obstructive sleep apnea.

## 2. Materials and Methods

This prospective observational study was conducted at the Department of Neurology, University Hospital Centre Osijek, from September 2019 to May 2022. Sixty adult patients aged 18–65 years with newly diagnosed moderate to severe obstructive sleep apnea (OSA) were enrolled. All participants provided written informed consent after being fully informed about the study objectives, procedures, data confidentiality, and their rights, including voluntary participation and withdrawal without consequences for medical care.

### 2.1. Inclusion and Exclusion Criteria

Inclusion criteria were age 18–65 years, both sexes, newly diagnosed moderate to severe OSA, normal hearing thresholds (≤25 dB HL at 500–4000 Hz), and good adherence to CPAP therapy.

Exclusion criteria included prior OSA treatment, mild OSA, other sleep disorders, pulmonary disease, substance or alcohol abuse, and poor CPAP adherence (<4 h/night on <70% of nights). Medical, neurological, and psychiatric conditions known to affect cognitive function were also excluded, including uncontrolled arterial hypertension, diabetes mellitus with end-organ complications, major depressive disorder, prior cerebrovascular disease, neurodegenerative disorders, and the use of psychoactive medications.

### 2.2. Baseline Assessments

Baseline evaluation included neurological examination, anthropometric measurements (weight, height, BMI), and administration of the Epworth Sleepiness Scale (ESS), STOP-Bang, and Berlin questionnaires. Cognitive function was assessed using the Montreal Cognitive Assessment (MoCA), and hearing thresholds were determined by pure-tone audiometry. MoCA was selected as the cognitive assessment tool because it provides higher sensitivity and specificity for detecting OSA-related mild cognitive impairment compared with the Mini-Mental State Examination (MMSE) [23]. Previous studies have shown that MoCA correctly identifies 72–81% of OSA patients with MCI, with specificity between 72% and 86%, outperforming the MMSE, which exhibits limited sensitivity and poor diagnostic accuracy in OSA populations [24].

### 2.3. Polysomnography

OSA diagnosis was confirmed by overnight computerized polysomnography (PSG) using the Alice 6 Diagnostic Sleep System (Philips Respironics, Murrysville, PA, USA). The PSG protocol included a 16-channel EEG setup (10–20 system), electrooculography (EOG), electromyography (chin and tibialis anterior), electrocardiography (lead II), and respiratory monitoring (thoracoabdominal plethysmography, nasal/oral airflow sensors, and pulse oximetry). Sleep scoring and event detection followed the American Academy of Sleep Medicine (AASM) version 2.4 using Sleepware *G3* software (v3.9.1). Recorded parameters included total sleep time, sleep efficiency, sleep stage distribution, apnea–hypopnea index (AHI), oxygen desaturation index (ODI), and periodic limb movement index (PLMI).

### 2.4. CPAP Therapy and Compliance

Following diagnosis, CPAP titration was performed using an auto-adjusting device that increased inspiratory pressure as required and reduced expiratory pressure for comfort. Compliance data (usage hours, residual AHI, pressure settings) were extracted from the device’s SD card. CPAP adherence was assessed using the Centers for Medicare and Medicaid Services (CMS) definition, which considers patients adherent if the device is used for at least 4 h per night on at least 70% of monitored nights [25]. Recorded parameters included residual AHI, average nightly use, and mean applied pressure.

### 2.5. Event-Related Potentials (ERPs)

Cognitive ERPs were recorded using a Medelec Synergy EMG/EP system (VIASYS Healthcare Inc. NeuroCare Group, 5225-2 Verona Rd., Madison, WI, USA) with an auditory “oddball” paradigm. Frequent standard tones (1000 Hz) and rare target tones (2000 Hz) were presented binaurally (80 dB SPL, 50 ms). Electrode impedances were maintained < 5 kΩ. EEG was recorded at Fz, Cz, and Pz, referenced to linked mastoids (A1, A2). Filter settings were 0.1–70 Hz, with a 50 Hz notch filter. Epochs were recorded from 0 to 700 ms post-stimulus. P300 latency and amplitude were analyzed.

### 2.6. Audiometry and Additional Measurements

Pure-tone audiometry was performed at the Department of Otorhinolaryngology using a GSI AudioStar Pro audiometer (Grason-Stadler, 7625 Golden Triangle Drive, Suite F, Eden Prairie, MN, USA) in a soundproof booth to exclude participants with hearing loss.

Blood pressure was measured before PSG using a Ri-san^®^ manual sphygmomanometer (Rudolf Riester GmbH, Jungingen, Njemačka), and fasting capillary glucose was measured the morning after PSG using an Accu-Chek Performa glucometer (Roche Diagnostics, Basel, Switzerland).

### 2.7. Follow-Up

At the 3-month follow-up, participants repeated the ESS, MoCA, and P300 assessments. CPAP adherence and residual respiratory events were reassessed.

### 2.8. Statistical Analysis

To detect a medium effect size (Cohen’s d = 0.5) in pre–post differences after three months of CPAP therapy, with a significance level of α = 0.05 and a statistical power of 0.95, a minimum sample size of 54 participants was required. As the study included 60 participants, it was adequately powered to detect such differences. Missing values were not replaced, and all analyses were performed on complete cases.

Categorical variables were summarized as absolute and relative frequencies, and differences before and after CPAP were analyzed using the marginal homogeneity test. Normality of continuous variables was tested using the Shapiro–Wilk test. Continuous data were expressed as medians and interquartile ranges. Paired comparisons were performed using the Wilcoxon signed-rank test, and between-group differences using the Mann–Whitney U or Kruskal–Wallis test (Conover post hoc test). Associations were examined using Spearman’s rank correlation Rho. Predictors of cognitive outcomes were identified using stepwise multivariate logistic regression. Receiver operating characteristic (ROC) analysis was applied to determine optimal cut-off values. All *p*-values are two-tailed. The significance level was set at alpha = 0.05. All statistical analyses were performed using MedCalc^®^ v20.100 (MedCalc Software Ltd., Ostend, Belgium) and IBM SPSS Statistics v23 (IBM Corp., Armonk, NY, USA).

## 3. Results

A total of 60 participants were included in the study, of whom 48 (80%) were male. The median age was 51 years (interquartile range [IQR], 45–58 years), with ages ranging from 29 to 65 years. The anthropometric characteristics and polysomnographic parameters of the participants are summarized in Table 1 and Table 2, respectively.

The Apnea–Hypopnea Index (AHI), an indicator of obstructive sleep apnea severity, ranged from 23.5 to 89.6 events/hour before therapy and significantly decreased after CPAP application, ranging from 2.0 to 4.7 events/hour (Wilcoxon test, *p* < 0.001) (Table 3).

**Figure 1:**
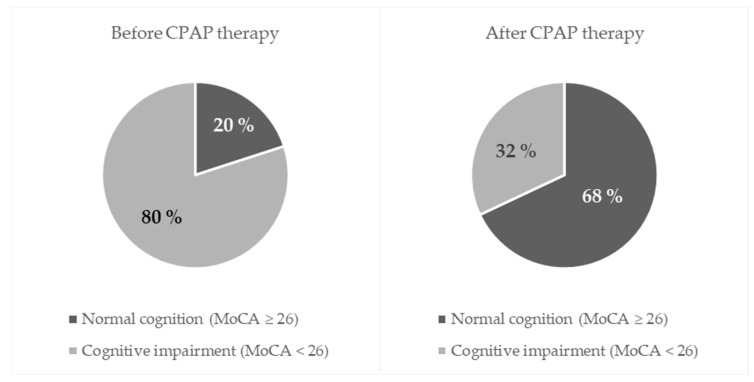
Prevalence of cognitive impairment before and after CPAP therapy.

Cognitive function was assessed using the MoCA test. Before therapy, 48 participants (80%) demonstrated cognitive impairment (MoCA < 26). After CPAP therapy, cognitive impairment was observed in only 19 participants (32%), corresponding to a 48-percentage-point reduction. This change was statistically significant (McNemar–Bowker test, *p* < 0.001). Among discordant pairs, 29 participants improved and none worsened, yielding a paired odds ratio of 59.0 (95% CI: 3.6–975), indicating a pronounced treatment effect (Table 4, Figure 1).

Significant post-therapy improvements were observed in several MoCA domains, including executive function (*p* < 0.001), attention (*p* < 0.001), delayed recall (*p* < 0.001), and total MoCA score (*p* < 0.001) (Table 5).

Baseline AHI showed significant correlations with cognitive improvement in two MoCA domains: visuospatial/executive function (rho = 0.305, *p* = 0.02) and language (rho = −0.333, *p* = 0.009). No significant correlations were observed for other domains or for the overall MoCA score, indicating that the cognitive benefits of CPAP were independent of baseline OSA severity (Table 6).

Cognitive electrophysiological function, assessed by event-related potentials (P300), showed significant post-therapy changes. After 3 months of CPAP, P300 latency shortened from a median of 339.5 ms (IQR 325.5–359.0) to 313.5 ms (304.0–325.5); Hodges–Lehmann (HL) median difference −22.0 ms (95% CI −27.5 to −17.5; *p* < 0.001). P300 amplitude increased from 9.75 μV (6.25–14.10) to 10.30 μV (7.25–14.65); HL 0.55 μV (95% CI −0.50 to 1.50; *p* = 0.25). Earlier components showed a modest latency shortening for P1 (39.0 ms [25.5–48.5] to 31.0 ms [26.0–40.0]; HL −5.5 ms; *p* = 0.01), with no significant changes for N1 (95.0 ms [90.0–108.0] to 94.0 ms [85.5–103.5]; HL −1.5 ms, 95% CI −5.0 to 2.5; *p* = 0.42) or P2 (181.0 ms [171.0–196.5] to 180.0 ms [165.0–195.0]; HL 0.0 ms, 95% CI −5.5 to 5.5; *p* = 0.99). A later negative component (labelled “N1” in the data table) showed no significant change (235.5 ms [225.0–262.5] to 236.5 ms [220.5–265.0]; HL −2.0 ms, 95% CI −9.5 to 4.5; *p* = 0.47) (Table 7).

Overall, CPAP therapy resulted in a significant improvement in both cognitive (MoCA) and electrophysiological (P300) measures, indicating partial neurocognitive recovery after three months of treatment.

## 4. Discussion

The present study demonstrated significant cognitive improvement following three months of continuous positive airway pressure (CPAP) therapy in patients with moderate and severe obstructive sleep apnea (OSA). Although MoCA scores increased from a median of 23 to 27, indicating clinically meaningful recovery, this improvement should not be interpreted as full normalization of cognitive function. A substantial proportion of patients continued to exhibit residual deficits at follow-up, which is expected given the short treatment duration and the chronic nature of OSA-related neural injury [26]. Improvements were most prominent in executive function, attention, and delayed recall—domains particularly vulnerable to intermittent hypoxemia and sleep fragmentation. In addition, a significant reduction in P300 latency and a mild, non-significant increase in amplitude were observed, suggesting partial electrophysiological recovery of cognitive processing.

Several mechanisms likely underlie these improvements. Restoration of nocturnal oxygenation decreases oxidative stress, mitochondrial dysfunction, and neuroinflammation—pathways strongly implicated in the disruption of hippocampal, parietal, and prefrontal networks [23,27]. Reversal of intermittent hypoxemia may promote synaptic plasticity and restore neural signalling within executive and attentional circuits [27].

In our study, prior to CPAP therapy, 80% of participants exhibited cognitive impairment (MoCA < 26), while after three months of treatment only 32% remained cognitively impaired. These results align with previous studies demonstrating that CPAP therapy can improve neurocognitive functioning in patients with OSA. Gad et al. reported significant post-treatment improvements in executive function, attention, naming, and delayed recall, while Gemici et al. found a strong association between total MoCA scores and apnea–hypopnea index (AHI), with cognitive improvement evident after three months of CPAP use but not after one day [28,29]. Similarly, Bucks et al. demonstrated significant gains in memory and attention following CPAP therapy [4]. Collectively, these findings suggest that the beneficial effects of CPAP on cognition require sustained treatment to counteract the cumulative impact of hypoxemia and sleep fragmentation.

In addition to global improvements in cognitive performance, our findings indicate that the extent of cognitive recovery following CPAP therapy was largely independent of baseline OSA severity. Baseline AHI showed no significant association with changes in the overall MoCA score, suggesting that patients across the severity spectrum may benefit similarly from treatment. Interestingly, modest but statistically significant correlations were observed in two specific cognitive domains: visuospatial/executive function (rho = 0.305, *p* = 0.02) and language (rho = −0.333, *p* = 0.009). These findings imply that certain cognitive processes—particularly those dependent on frontoparietal network integrity—may be more sensitive to the cumulative burden of sleep-disordered breathing. However, the absence of associations in other domains supports the notion that the observed improvements reflect generalized effects of restored nocturnal oxygenation and reduced sleep fragmentation rather than a severity-dependent gradient of cognitive recovery.

Our findings also confirm that patients with moderate and severe OSA typically present with lower baseline MoCA scores, consistent with mild cognitive impairment (MCI). Given that MCI often precedes dementia and is associated with an increased risk of neurodegenerative progression, OSA may represent a modifiable risk factor for dementia development. Indeed, previous population-based research has shown that between 6% and 20% of individuals over 60 years have MCI, and up to 27% of them also have OSA, reinforcing this relationship [30,31,32].

The P300 component of ERPs serves as an electrophysiological correlate of cognitive performance. Its amplitude reflects attentional engagement, while latency indicates the speed of stimulus evaluation and information processing. In our study, P300 latency significantly decreased after three months of CPAP therapy (from 339.5 ms to 313.5 ms), suggesting improved cortical responsiveness and faster cognitive processing. Although amplitude increased modestly (from 9.75 µV to 10.3 µV), the change was not statistically significant. Similar findings were reported by Ak et al. and El-Gharib et al., who also observed shortened P300 latencies and mild amplitude increases after three months of CPAP treatment [33,34]. Martins et al. proposed that reduced P300 amplitudes in untreated OSA patients reflect auditory memory deficits, which tend to normalize with effective therapy [15].

Despite consistent trends toward improvement, not all studies have demonstrated full cognitive recovery following CPAP therapy. Vakulin et al. suggested that residual cognitive deficits may persist in some patients, particularly in severe OSA, possibly due to irreversible hypoxic injury or suboptimal treatment adherence [35]. Differences in study design, sample size, and the sensitivity of cognitive tests likely contribute to the heterogeneity of published findings.

The present study has certain limitations, including a relatively small sample size and the use of a single neuropsychological test (MoCA). Future research should include larger cohorts, longer follow-up periods, and a more detailed neuropsychological battery, along with advanced ERP analyses, to better elucidate the mechanisms of cognitive recovery in OSA.

In summary, our results indicate that effective CPAP therapy leads to measurable cognitive recovery in patients with moderate and severe OSA, reflected in both improved MoCA scores and reduced P300 latency. These findings support the role of CPAP not only in restoring normal breathing during sleep but also in reversing the cognitive consequences of untreated OSA. These insights emphasize the need for routine cognitive screening in OSA management and support the integration of neurocognitive assessment into clinical sleep medicine protocols.

## 5. Conclusions

This study demonstrated that continuous positive airway pressure (CPAP) therapy promotes significant neurocognitive recovery in patients with moderate-to-severe obstructive sleep apnea (OSA). After three months of treatment, participants exhibited marked improvements in executive function, attention, and delayed recall, accompanied by a significant reduction in P300 latency, indicating enhanced cognitive processing speed and neural efficiency.

These findings underscore the importance of early diagnosis and consistent CPAP adherence to prevent or potentially reverse cognitive decline in patients with OSA. The observed improvements highlight the potential of combining neuropsychological and electrophysiological assessments, such as MoCA and P300, to monitor treatment efficacy and cognitive outcomes in sleep medicine practice.

Future studies with larger cohorts, extended follow-up periods, and multimodal neuroimaging should further elucidate the mechanisms underlying cognitive recovery and determine whether CPAP therapy can fully restore brain function in OSA.

## Figures and Tables

**Table 1:** Anthropometric characteristics of the participants.

Anthropometric Characteristic	Median (IQR)	Minimum–Maximum
Body Weight (kg)	105 (92–135)	75–175
Body Height (cm)	176 (170–183)	160–196
BMI (kg/m^2^)	33.60 (30.73–42.08)	26.20–63.50
Heart Rate (bpm)	69 (62.6–75.4)	46–96
Systolic Blood Pressure (mmHg)	130 (122–135)	110–150
Diastolic Blood Pressure (mmHg)	80 (75–80)	70–95
Blood Glucose Level (mmol/L)	5.5 (5.1–5.9)	4.5–7.6

Abbreviations: IQR—Interquartile Range, BMI—Body Mass Index.

**Table 2:** Polysomnographic features of participants.

Sleep Parameters	Median (IQR)	Minimum–Maximum
Time in bed (min)	466.35 (443.70–501.45)	369–776.30
Total Sleep Time (min)	405.45 (367–446.88)	300.50–570
Stage N1 (%)	12.20 (8.05–15.3)	1–34.60
Stage N2 (%)	57.65 (51.78–64.2)	25–82.80
Stage N3 (%)	17.50 (12.73–25.5)	2–33
NREM sleep (%)	88.70 (83.73–91.38)	26.50–99.10
REM sleep (%)	10.90 (7.88–16.13)	0.90–24.60
Sleep Onset Latency (min)	11.05 (5.93–24.35)	0.40–109.50
REM Latency (min)	121.25 (94.25–195.3)	53–426
WASO (%)	31.45 (10.20–62.70)	0.10–241.40
Number of Sleep Cycles	4 (3–4)	1–6
Sleep Efficiency (%)	86.30 (81.90–92.65)	23.50–99.60
Mean Saturation (%)	91 (89–93)	74–97
Lowest Saturation (%)	74.50 (64.25–80)	35–90
ODI (events/hour)	59.10 (42.90–75.82)	9.51–133.5

Abbreviations: IQR—Interquartile Range, NREM—non-rapid eye movement; REM—rapid eye movement; WASO—wake after sleep onset; ODI—oxygen desaturation index.

**Table 3:** AHI before and after CPAP therapy.

	Median (IQR)		Hodges-Lehmann Median Difference	95% Confidence Interval	* *p*-Value
	Before CPAP Therapy	After CPAP Therapy			
AHI (events/h)	56.4 (23.5–71.7)	3.0 (2.0–4.8)	−53.2	−58.6 to −48.2	**<0.001**

* Mann–Whitney U test. Abbreviations: AHI—apnea-hypopnea index (events per hour); CPAP—continuous positive airway pressure therapy. Bold values denote statistical significance.

**Table 4:** MoCA before and after CPAP therapy.

		Number (%) of Participants Before CPAP Therapy			* *p*-Value
		Normal Cognition (MoCA ≥ 26)	Cognitive Impairment (MoCA < 26)	Total	
After CPAP therapy	Normal cognition (MoCA ≥ 26)	12	29	41 (68)	**<0.001**
	Cognitive impairment (MoCA < 26)	0	19	19 (32)	
	Total	12 (20)	48 (80)	60 (100)	

* McNemar–Bowker test; Effect size: paired OR = 59.0 (95% CI: 3.6–975); Abbreviations: CPAP—continuous positive airway pressure therapy; MoCA—Montreal Cognitive Assessment; Bold values denote statistical significance.

**Table 5:** Differences in specific cognitive domains and the overall MoCA score before and after CPAP therapy.

	Median (IQR)		Hodges-Lehmann Median Difference	95% Confidence Interval	* *p*-Value
	Before CPAP Therapy	After CPAP Therapy			
Visuospatial/Executive Function	3 (2–4)	5 (3–5)	1.5	1 to 1.5	**<0.001**
Naming	3 (3–3)	3 (3–3)	0	0 to 0	0.06
Attention	5 (4–6)	6 (5–6)	0.5	0.5 to 1	**<0.001**
Language	3 (2–3)	3 (3–3)	0	0 to 0	0.06
Abstraction	2 (2–2)	2 (2–2)	0	0 to 0	0.06
Delayed Recall	4 (2–5)	5 (4–6)	1.5	1 to 2	**<0.001**
Orientation	6 (6–6)	6 (6–6)	0	0 to 0	-
Overall MoCA score	23 (20–25)	27 (25–28)	4	3 to 5	**<0.001**

* Mann–Whitney U test; Abbreviations: IQR—Interquartile Range; CPAP—continuous positive airway pressure therapy; MoCA—Montreal Cognitive Assessment. Bold denotes statistical significance.

**Table 6:** Correlation between baseline OSA severity (AHI) and improvement in cognitive function after CPAP therapy.

	Spearman’s Rho (*p* Value)
	Correlation Between Baseline AHI and Δ(Post–Pre) MoCA Scores
ΔVisuospatial/Executive Function	**0.305 (0.02)**
ΔNaming	0.008 (0.95)
ΔAttention	0.116 (0.38)
ΔLanguage	**−0.333 (0.009)**
ΔAbstraction	0.145 (0.27)
ΔDelayed Recall	−0.053 (0.69)
ΔOrientation	-
ΔOverall MoCA score	0.105 (0.42)

Bold denotes statistical significance.

**Table 7:** Differences in ERP P300 before and after CPAP therapy.

	Median (IQR)		Hodges-Lehmann Median Difference	95% Confidence Interval	* *p*-Value
	Before CPAP Therapy	After CPAP Therapy			
P1 wave latency (ms)	39 (25.5–48.5)	31 (26–40)	−5.5	−10.5 to −9.0	**0.01**
N1 wave latency (ms)	95 (90–108)	94 (85.5–103.5)	−1.5	−5 to 2.5	0.42
P2 wave latency (ms)	181 (171–196.5)	180 (165–195)	0	−5.5 to 5.5	0.99
N2 wave latency (ms)	235.5 (225–262.5)	236.5 (220.5–265)	−2	−9.5 to 4.5	0.47
P300 wave latency (ms)	339.5 (325.5–359.0)	313.5 (304–325.5)	−22	−27.5 to −17.5	**<0.001**
P300 wave amplitude (μV)	9.75 (6.25–14.1)	10.3 (7.25–14.65)	0.55	−0.5 to 1.5	0.25

* Mann–Whitney U test; Abbreviations: CPAP—continuous positive airway pressure therapy, IQR—interquartile range. Bold denotes statistical significance.

## Data Availability

The original contributions presented in this study are included in the article. Further inquiries can be directed to the corresponding author.

## Abbreviations

The following abbreviations are used in this manuscript:

AHI	Apnea-hypopnea index
BAER	Brainstem auditory evoked response
CPAP	Continuous positive airway pressure
ERP	Event-related potentials
OSA	Obstructive sleep apnea
